# Milk β-hydroxybutyrate metrics and its consequences for surveillance of hyperketonaemia on commercial dairy farms

**DOI:** 10.3389/fvets.2023.1272162

**Published:** 2023-11-08

**Authors:** Elise De Jong, Angelique Rijpert-Duvivier, Hendrik Veldman, Wilma Steeneveld, Ruurd Jorritsma

**Affiliations:** ^1^Northern Country Animal Care, Cobram, VIC, Australia; ^2^Elanco Benelux, Utrecht, Netherlands; ^3^Delaval Benelux, Steenwijk, Netherlands; ^4^Department of Population Health Sciences, Faculty of Veterinary Medicine, Utrecht University, Utrecht, Netherlands

**Keywords:** hyperketonaemia, ketosis, dairy cows, monitoring, testing

## Abstract

Dairy cows that are unable to adapt to a change in their metabolic status are at risk for hyperketonaemia (HK). Reported HK herd level prevalences range a lot and we hypothesized that this is partly due to differences in used tests and monitoring protocols. Insights in milk β-hydroxybutyrate (BHB) metrics can potentially explain why the reported incidences or prevalences vary between test strategies. Automated collection and repeated analyses of individual milk samples with the DeLaval Herd Navigator^™^ (HN) provides real-time data on milk BHB concentrations. We aimed to use that information to gain insight in BHB metrics measured in milk from 3 to 60 days in milk (DIM). Using different cut-offs (0.08, 0.10 and 0.15 mmol/L), 5 BHB metrics were determined. Furthermore, the impact of 4 arbitrary test protocols on the detected incidence of HK was assessed. We used HN data of 3,133 cows from 35 herds. The cumulative incidence of HK between 3 and 60 DIM varied between 30.5 and 76.7% for different cut-off values. We found a higher HK incidence for higher parity cows. The first elevated BHB concentrations were roughly found between one and two weeks after calving. For higher parity cows the maximum BHB concentrations were higher, the onset of HK was earlier after calving, and the number of episodes of HK was higher. It appeared that the sensitivity of a HK test protocol can be increased by increasing the testing frequency from once to twice a week. Also extending the number of days of the test window from 4–14 to 4–21 days enhances the chance to find cows experiencing HK. In conclusion, HN data provided useful insights in milk BHB metrics. The chosen cut-off value had a large effect on the reported metrics which explains why earlier reported incidences or prevalences vary such a lot. Differences in test period and sample selection also had a large impact on the observed HK incidence. We suggest to take this in consideration while evaluating whether HK is an issue on farm level and use a uniform protocol for benchmarking of HK between farms.

## Introduction

The transition period is a critical period for a dairy cow as she needs to adjust her feed intake and energy metabolism to support the high milk production (1). As the increase in energy intake lags behind on the high and prioritized energy demand for milk production, dairy cows are typically characterized by a negative energy balance in early lactation. Cows that are unable to adapt to this change in their metabolic status are at risk for hyperketonaemia (HK), which can be divided into clinical ketosis and subclinical ketosis. Clinical ketosis manifests as weight loss, decrease in appetite, a decrease in milk production, and sometimes neurological signs. Subclinical ketosis is defined as a surplus of ketone bodies (acetone, acetoacetate and β-hydroxybutyrate (BHB)) in blood, but without clinical signs. Cows with HK have a higher risk to be removed from the herd, produce less milk, have a higher odds to develop diseases, have a lower reproductive performance (2, 3), and cause economic losses (4–6).

Reported HK herd level point prevalences range from 0 to 80% (7) and from 11.2 to 36.6% (3, 8, 9). The large variation is likely due to differences between farms and regions, but it is also possible that the small number of cows at-risk may inflate or deflate this percentage. Moreover, repeated sampling will increase the number of HK detected cows, which can be reported as early lactational prevalence or incidence as cows are assumed to have no HK at parturition (10). The range of reported incidences is also rather large (11).

Measurement of BHB in blood is considered the golden standard for HK and most studies use a BHB-concentration of ≥1.2 mmol/L in blood to define HK. However, monitoring BHB in milk is also commonly used and shows good correlations with the golden standard (12). Advantages of milk sampling over blood sampling includes that it is not invasive, it can be automated, and it reflects BHB concentration for a longer time frame. Automated collection and repeated analyses of milk samples is currently commercially available with the DeLaval Herd Navigator^™^ (HN). The HN analyses milk samples of individual cows and provides also real-time data on BHB-concentration for HK detection. BHB concentration in milk is measured at least daily from 3 to 20 days in milk (DIM) and at least once every 4 days from 20 to 60 DIM. The advantage of repeated sampling of BHB in milk is that it can provide insights in BHB metrics at cow level, such as the duration of elevated BHB, the onset of high BHB and the fluctuations of the BHB concentration. Only few studies investigated BHB metrics (mostly in blood) and focused on the associations with diseases, culling, and fertility (2, 13). Other aspects, such as the number of episodes of HK, have not been reported yet. Insights in the milk BHB metrics can potentially explain why the reported incidences or prevalences vary between test strategies and are useful to interpret test results.

The main aim of this study was therefore to provide insight in BHB metrics using HN data on BHB measured in milk from 3 to 60 DIM. Using different cut-off values of milk BHB, we focused on the following 5 metrics: cumulative incidence of elevated BHB, onset of elevated BHB, maximum BHB concentration, duration of elevated BHB, and the number of episodes of HK. In addition, 4 test protocols are defined that are feasible in practice for the data from 4 to 14 or 21 DIM. The second aim was to evaluate the performance of the test protocols by determining the obtained cumulative incidences.

## Materials and methods

### Available data

DeLaval (DeLaval Inc., Tumba, Sweden) provided historical data from the Lattec I/S (Lattec I/S, Hillerød, Sweden) database of 41 herds from 1st of July 2018 till 30 June 2019 on parity, calving date, culling-date, BHB-measurements in milk from 3 to 60 DIM, and daily milk yield. The farms were located in Belgium, Luxembourg or Netherlands.

The BHB measurements were executed by the HN. The HN analyses milk samples of individual cows and provided real-time data for ketosis detection. An inline sampler inside the AMS automatically took a representative sample of milk from an individual cow during the milking process. HN analysed this sample, and measured BHB-concentration for ketosis detection. In the first 20 DIM, BHB was measured at least once per day, but sometimes twice or thrice. Between 20–60 DIM, the measuring frequency was based on the previous BHB measurement, but at least every 4 days (14). BHB data included both raw and algorithm smoothed values of BHB measurements, but in this study only raw BHB values were used to reflect the actual BHB concentrations of cows.

### Definitions of milk BHB metrics

To investigate milk BHB metrics up to 60 DIM, we used three BHB cut-off values (0.08, 0.10, and 0.15 mmol/L). The cut-off of 0.08 mmol/L was chosen because the HN uses this level to alert farmers that a cow has HK. We selected the cut-off of 0.10 mmol/L as this level was found discriminating in another study on HK testing in milk (15). Finally, a much higher level of 0.15 mmol/L was added to investigate how this would stretch the selected metrics. Definitions of the milk BHB metrics are provided in Table 1.

**Table 1:** Definition of the milk β-hydroxybutyrate (BHB) metrics as measured with the Herd navigator.

Milk BHB metrics	Explanation
Cumulative incidence^a^	Percentage of cows with at least one measurement with a BHB concentration above the cut-off value during 3–60 days in milk (DIM)
Onset^a^	First DIM of a BHB value above the cut-off value
Maximum	Maximum BHB concentration between 3 and 60 DIM
Consecutive duration^a^^,^^b^	Number of days of the longest period with consecutive BHB concentrations above the cut-off value
Episodes of HK^a^	Occasion at which the BHB concentration rose again to above the cut-off value after a measurement below this value

a Calculated for three different BHB cut-off values (0.08, 0.10, and 0.15 mmol/L).

b In case one or more consecutive missing value(s) was followed by a negative value, it was considered negative while they were considered positive when followed by a positive value.

### Tested protocols

To facilitate the discussion about the effect of the BHB metrics on testing methods, we arbitrary defined 4 possible test protocols, using the 0.10 mmol/L cut-off level. To avoid possible effects of the sampling strategy of the HN on the comparison of the protocols, we decided to use data of the first 20 DIM only. The protocols differed in the used test window and the selection of the samples within the windows. Both used windows are commonly used on farms in the participating countries. Definitions are provided in Table 2.

**Table 2:** Mean herd cumulative incidence (and range between brackets) of cows with milk β-hydroxybutyrate (BHB) above 0.10 mmol/L for 5 different monitoring protocols.

Protocol	Test window (DIM)	Sample selection	Cumulative incidence (%)
A	4–21	Weekly, starting at a random day in the week	21.2 (4.2–48.1)
B	4–14	Weekly, starting at a random day in the week	14.2 (2.8–34.6)
C	4–14	Once, at a random selected DIM per cow	10.4 (13.9–23.1)
D	4–14	Twice weekly (every 3 or 4 days), starting at a random day in the week	20.8 (5.6–48.1)
Reference protocol		No sample selection: all samples 3–60 DIM	52

### Data editing

Only herds that used an HN at least since January 2017 were considered eligible because this ensured that the farmers used the HN for their cow management for at least 1.5 year. Consequently, 3 herds were excluded. An additional 3 herds were excluded because their dataset was not complete. We started therefore with a dataset containing 35 herds with a total of 149,249 BHB measurements and a dataset containing 4,848 cows, which were merged using herd-specific and unique cow-ID’s. BHB-measurements before 3 DIM or after 60 DIM (*n* = 1,005) were excluded as they fall outside the HN BHB test protocol (14). Consequently, 148,244 BHB-measurements remained in the dataset. Milk BHB-values ranged between 0.0003 and 1.19 mmol/L. Data-editing further included aggregating information per unique cow and parity combination for: herd, unique cow-number, parity, calving date, first date with a BHB-measurement, lowest DIM with a BHB-measurement, last date with a BHB-measurement, highest DIM with a BHB-measurement, number of BHB-measurements, maximum BHB concentration, and BHB-measurements for each DIM between 3 and 60 DIM. In case there were multiple BHB-measurements for a cow on the same DIM, 1 BHB measurement was randomly selected.

In a next step, we excluded 1,715 cows as the cows had no BHB measurements (*n* = 213), were removed from the herd within 60 DIM (*n* = 159), the recorded calving date did not match with the DIM (*n* = 37), they had less than 16 BHB measurements between 3 and 20 DIM or less than 9 BHB measurements between 21 and 60 DIM (*n* = 547), did not have a first BHB measurement within the first 5 DIM and did not have a last BHB measurement after 55 DIM (*n* = 24) which is less than expected given the HN test protocol. Furthermore, we removed cows that had not a BHB measurement on the randomly selected date according to the test protocols that are presented in Table 2 (*n* = 735). This resulted in a final dataset with 3,133 cows, who’s records were used in all analyses.

From the 35 herds, 30 were located in Netherlands, 4 herds in Belgium, and 1 in Luxembourg. Mean number of included cows per herd after data editing was 89 (range = 14–290). The mean proportion of cows for parity 1, 2, and ≥3 was, respectively, 25.4% (range = 7.1–40.6), 27.7% (range = 9.1–71.4), and 46.9% (range = 27.2–70.4). Mean milk production per day per herd was 29.7 kg milk (range = 21.2–42.3).

Data editing and descriptive statistics were performed with RStudio and the packages dplyr, tidyr, and ggplot2 (Version 1.2.1335^©^ 2009–2019 RStudio, Inc.).

## Results

### Cumulative incidence

The highest cumulative incidence of HK was found when the lowest BHB cut-off was used (0.08 mmol/L, 76.7%). Highest cumulative incidences were found in higher parity cows (see Table 3).

**Table 3:** Overall and parity specific cumulative incidence per herd (*N* = 35) between 3–60 days in milk at different milk β-hydroxybutyrate (BHB) cut-off values based on a dataset including 3,133 cows.

	Milk BHB cut-off value (mmol/L)		
	0.08	0.10	0.15
Overall cumulative incidence (%)	76.7 (53.8–93.9)	52.0 (28.8–78.0)	30.5 (11.1–60.9)
parity 1 (%)	61.1 (27.3–100)	35.5 (0.0–85.7)	19.6 (0.0–85.7)
parity 2 (%)	77.4 (45.8–100)	47.7 (16.7–91.7)	24.6 (0.0–70.6)
parity ≥3 (%)	84.3 (62.5–100)	63.0 (33.3–90.0)	39.8 (12.5–80.0)

### Onset

The median first day of elevated BHB for the different cut-off values within the herd is presented in Table 4. Depending on the used cut-off value, the median first day of HK increased from 8.9 DIM for 0.08 mmol/L to 15.8 DIM for 0.15 mmol/L. The first day of HK was earlier in lactation for higher parity cows.

**Table 4:** Herd level (*N* = 35) median first day of milk β-hydroxybutyrate (BHB) concentration reaching or exceeding three different cut-off values for different parities (range between brackets).

	Milk BHB cut-off value (mmol/L)		
	0.08	0.10	0.15
All parities	8.9 (6–19.5)	11.9 (8.0–24.5)	15.8 (8.0–27.0)
Parity 1	13.9 (7–35)	16.9 (5.0–51.5)^a^	22.0 (6.0–44.0)^b^
Parity 2	10.6 (5–29)	13.1 (6.0–32.0)	18.8 (4.0–43.0)^a^
Parity ≥3	7.9 (5–19)	11.0 (6.0–25.5)	15.5 (11.0–38.0)

a Based on 33 herds.

b Based on 31 herds.

### Maximum BHB value

The median of all maximum BHB-concentrations per herd was 0.11 mmol/L. We found that the median of the maximum BHB concentration was lower for cows with parity 1 (0.09 mmol/L) compared to parity 2 (0.10 mmol/L), and cows with parity 3 or higher (0.13 mmol/L).

### Consecutive duration of elevated BHB period and episodes of HK within that period

Table 5 shows the consecutive duration of HK for different cut-off values. The longest consecutive duration of HK decreases at higher BHB cut-off values, except for parity 1. The proportion of cows per herd with one or more episode of HK is the highest for cut-off value 0.08 mmol/L and parity ≥3 and decreases at higher cut-off value and lower parity.

**Table 5:** Mean (and range between brackets) consecutive duration and proportion of numbers of hyperketonaemia (HK) episodes with different milk β-hydroxybutyrate (BHB) cut-off value (mmol/L) at herd level for 35 herds.

	Milk BHB cut-off value (mmol/L)		
	0.08	0.10	0.15
Median consecutive duration HK (days)^a^	4.1 (1.0–9.0)	3.4 (1.0–8.5)	3.0 (1.0–6.5)
Parity 1	3.3 (1.0–20.0)	3.3 (1.0–17.0)^b^	5.0 (1.0–27.0)^c^
Parity 2	3.0 (1.0–7.0)	3.2 (1.0–16.0)	2.7 (1.0–10.0)^b^
Parity ≥3	6.0 (2.0–13.0)	4.3 (1.0–10.0)	3.1 (1.0–6.0)
0 HK episodes (%)	24.7 (7.3–48.0)	49.4 (23.6–70.4)	71.2 (4.4–90.1)
Parity 1	40.2 (0.0–81.0)	64.9 (22.2–90.5)	81.3 (22.2–100)
Parity 2	24.1 (0.0–56.3)	53.6 (18.2–81.3)	76.1 (45.5–100)
Parity ≥3	15.9 (0.0–38.8)	37.9 (13.0–61.7)	62.0 (26.1–90.2)
1 HK episodes (%)	18.9 (9.8–31.8)	16.9 (5.5–27.3)	11.6 (4.4–19.7)
Parity 1	22.4 (4.8–42.3)	15.3 (0.0–33.3)	9.0 (0.0–25.0)
Parity 2	20.3 (0.0–60.0)	16.9 (0.0–30.0)	11.0 (0.0–22.7)
Parity ≥3	15.8 (6.5–50.0)	18.2 (0.0–35.7)	13.4 (3.2–28.6)
2 HK episodes (%)	15.4 (10.7–27.3)	11.3 (5.3–22.0)	6.0 (0.0–19.2)
Parity 1	14.7 (0.0–66.7)	7.2 (0.0–25.0)	4.0 (0.0–22.2)
Parity 2	16.0 (0.0–47.4)	10.8 (0.0–23.1)	5.2 (0.0–20.0)
Parity ≥3	16.3 (8.3–25.0)	13.7 (5.0–27.6)	7.8 (0.0–22.2)
3 or more HK episodes (%)	41.1 (21.2–67.3)	22.4 (3.3–58.2)	11.2 (0.0–32.7)
Parity 1	22.7 (0.0–58.3)	12.7 (0.0–55.6)	5.7 (0.0–33.3)
Parity 2	39.6 (9.5–80.0)	18.7 (0.0–60.0)	7.8 (0.0–33.3)
Parity ≥3	51.6 (16.7–75.9)	30.2 (2.4–78.3)	16.8 (0.0–47.8)

a Longest consecutive period in days with samples above the cut-off value.

b Based on 33 herds.

c Based on 31 herds.

### Test protocols

Table 2 shows the cumulative incidence for the different test protocols, which shows that both the testing window and the number of samples taken within this window have an effect on the cumulative incidence.

## Discussion

We showed that the detected milk BHB cumulative incidence in the first 60 DIM depends heavily on the selected cut-off value as the cumulative incidence varies between 30.5 and 76.7%. Although the direction of these findings are obvious, we consider insights in the amount of variation valuable as this information is usually not provided in studies and has impact on the users (e.g., of the HN). The first elevated BHB concentrations were, dependent on the used cut-off value, roughly found between 1 and 3 weeks after calving. We illustrated that for higher parity cows, the HK incidence was higher, the maximum BHB concentrations were higher, the onset of HK was earlier after calving, and the number of HK episodes was higher compared to lower parity cows. Using different test protocols, we found that an increase in sampling frequency had a large impact on the calculated HK incidence (comparison protocol B and C), while extending the test window had a similar effect (comparison protocol A and B). We showed that a relative small decrease in BHB cut-off value resulted in a two or sometimes three times higher HK incidence and also affected other aspects of BHB metrics.

Studies performed in the past decade show different percentages of dairy cows with hyperketonaemia (1, 7, 8, 16). We illustrated in the current study that at least some of the differences may easily be explained by the selected cut-off value, sampling frequency, length of the test window and parity composition of the participating herd. As stated by others, these factors need to be taken in to account when comparing the percentage of cows with hyperketonaemia (12, 17, 18).

The median onset of 8.9 days at cut-off of 0.08 mmol/L was similar as found in other studies. Others found an average onset of hyperketonaemia of 9.06 ± 0.86 days at a cut-off of 1.2 mmol/L in blood samples (19) and a peak incidence of hyperketonaemia at 5 DIM (2). Previous studies showed that time of onset of hyperketonaemia in combination with the level of BHB at the onset of HK is an important indicator of individual cow performance (2, 20). In most of these studies though, cows were sampled over a shorter period of time. As illustrated in this study, this will decrease the cumulative incidence and could also affect the median onset of HK. Also, as the median onset of HK exceeds the 14 days threshold in particular when using the 0.15 mmol/L cut-off value, it seems necessary to continue BHB measurements for 3 weeks to measure individual cow performance.

Lower parity cows had a lower median of the maximum concentration of BHB and the narrowest range. This is in accordance with other studies where cows from third parity and onwards show higher BHB concentrations than second parity cows and heifers (1, 8, 21). The maximum BHB value reached could be an important HK metric in terms of HK severity. However, in this study, we were not able to and not aiming for assessing consequences of the maximum BHB concentration. We suggest that this may be used to evaluate in future studies.

The HK duration appeared not very sensitive for cut-off values and varied between 4.1 and 3.0 days with increasing cut-off values. This is similar to the results of another study where a median time of 5 days from first elevated BHB test to first test under the cut-off value of 1.2 mmol/L in blood was found (2). Furthermore, a median duration of 3–4 days was found with a test protocol once per 3 to 4 days in blood (16).

After the first episode of elevated BHB concentrations the median number of episodes of HK that cows experience is 2.2 at cut-off value of 0.08 mmol/L and decreases to 0.9 by increasing the cut-off to 0.15 mmol/L. While the proportion of cows per herd that experience no episodes of HK increases for higher cut-off values from 24.7 to 71.2 percent, the median number of episodes of HK is also higher for higher parity cows. This suggests that older cows have more difficulties to recover from the negative energy balance. Another explanation is that the incidence of type I vs. type II ketosis is different for older cows as others have suggested that the recovery for both types maybe not similar.

We evaluated the effect of the HK metrics in 4 arbitrary test protocols. It appeared that a higher testing frequency increases the sensitivity of a HK test protocol. The percentage found in protocol A in comparison with B and C shows that extending the number of days of the test window from 4–14 to 4–21 days also enhances the chance to find animals experiencing HK. Therefore, the high test-frequency and broad test window of the HN protocol obviously contribute to the much higher observed incidence compared with all alternative protocols. Others demonstrated that lactation stage at time of testing influences HK incidence (12).

Using the convenience sample of 35 herds we have likely selected farmers that have a special interest in disease monitoring and are probably driven to improve the health of transition cows. It is not known whether the farmers were motivated by, e.g., a lowered or an elevated number of diseases and assume that none of these groups are over-represented in our sample. As the size of herds and their production level is also comparable with other farms in the aforementioned countries, we think that the herds are a fair representation of all herds in the region. Although the removal of cows may affect the cumulative incidence, we felt that this was necessary for our analyses and justified as we had no reliable data on culling reasons.

The selection of a certain cut-off level and test protocol has an impact on the observed HK incidence. If a protocol is only used for the surveillance of BHB with the objective to detect an increase or decrease in incidence with the option to take interventions at the herd level, it is above all important to continuously use the same cut-off and the same protocol. Also for benchmarking between farms uniform methods are required, and this can probably best be achieved by automated testing. If a protocol is in particular used for the identification of cows that need a treatment it is important to investigate further whether for a given protocol a parity specific cut-off value is needed for the BHB concentration.

In conclusion, we showed that increasing the milk BHB cut-off value has an anticipated effect on the cumulative incidence of HK. Although the direction of the findings are obvious, we consider insights in the amount of variation in cumulative incidence of HK with different milk BHB cut-off values valuable as this information is usually not provided in studies and has impact on the users (e.g., of the HN). It also explains why earlier reported incidences or prevalences vary such a lot. Our data also suggest that the onset of HK frequently occurs after 14 DIM and that higher parity cows that recovered from a period of HK, often experience one or more additional episodes of HK. In addition, the 4 defined test protocols indicated that a difference in test period and sample selection has a large impact on the observed HK incidence. Therefore, to determine whether HK is an issue on farm level, the chosen protocol needs to be taken into consideration while benchmarking HK between farms also requires a similar monitoring protocol. Obtained insights can be used to start discussing whether surveillance and detection algorithms can use different cut-off values and test protocols for individual herds and cows.

## Data availability statement

The data analyzed in this study is subject to the following licenses/restrictions: data is owned by a commercial company. Requests to access these datasets should be directed to HV Hendrik.Veldman@delaval.com.

## Ethics statement

Ethical approval was not required for the studies involving animals in accordance with the local legislation and institutional requirements because we used data of measurements in milk, which was non invasively collected during the regular milking process. Written informed consent was obtained from the owners for the participation of their animals in this study.

## Author contributions

EJ: Data curation, Formal analysis, Methodology, Software, Writing – original draft. AR-D: Conceptualization, Funding acquisition, Resources, Supervision, Writing – review & editing. HV: Conceptualization, Funding acquisition, Resources, Supervision, Writing – review & editing. WS: Conceptualization, Supervision, Writing – review & editing, Methodology. RJ: Supervision, Writing – review & editing.
